# IL‐2mAb reduces demyelination after focal cerebral ischemia by suppressing CD8^+^ T cells

**DOI:** 10.1111/cns.13084

**Published:** 2018-11-15

**Authors:** Yu‐Xi Zhou, Xin Wang, Dan Tang, Yan Li, Ying‐Fu Jiao, Yu Gan, Xiao‐Ming Hu, Li‐Qun Yang, Wei‐Feng Yu, Ruth Anne Stetler, Pei‐Ying Li, Da‐Xiang Wen

**Affiliations:** ^1^ Department of Anesthesiology, Renji Hospital, School of Medicine Shanghai Jiaotong University Shanghai China; ^2^ State Key Laboratory of Oncogenes and Related Genes, Shanghai Cancer Institute, Renji Hospital Shanghai Jiao Tong University School of Medicine Shanghai China; ^3^ Pittsburgh Institute of Brain Disorders and Recovery University of Pittsburgh School of Medicine Pittsburgh Pennsylvania

**Keywords:** CD8^+^ T cell, demyelination, IL‐2 monoclonal antibody (JES6‐1), Stroke

## Abstract

**Aims:**

Demyelination, one of the major pathological changes of white matter injury, is closely related to T‐cell–mediated immune responses. Thus, we investigate the role of an IL‐2 monoclonal antibody (IL‐2mAb, JES6‐1) in combatting demyelination during the late phase of stroke.

**Methods:**

IL‐2mAb or IgG isotype antibody (0.25 mg/kg) was injected intraperitoneally 2 and 48 hours after middle cerebral artery occlusion (MCAO) surgery. Infarct volume, peripheral immune cell infiltration, microglia activation, and myelin loss were measured by 2,3,5‐triphenyte trazoliumchloride staining, immunofluorescence staining, flow cytometry, and Western blot. Intraperitoneal CD8 neutralizing antibody (15 mg/kg) was injected 1 day before MCAO surgery to determine the role of CD8^+^ T cells on demyelinating lesions.

**Results:**

IL‐2mAb treatment reduced brain infarct volume, attenuated demyelination, and improved long‐term sensorimotor functions up to 28 days after dMCAO. Brain infiltration of CD8^+^ T cells and peripheral activation of CD8^+^ T cells were both attenuated in IL‐2 mAb‐treated mice. The protection of IL‐2mAb on demyelination was abolished in mice depleted of CD8^+^ T cell 1 week after stroke.

**Conclusions:**

IL‐2mAb preserved white matter integrity and improved long‐term sensorimotor functions following cerebral ischemic injury. The activation and brain infiltration of CD8^+^ T cells are detrimental for demyelination after stroke and may be the major target of IL‐2mAb posttreatment in the protection of white matter integrity after stroke.

## 1 INTRODUCTION

Stroke is the leading cause of long‐term disability and mortality, posing an enormous burden on patients and their community across the world.^1^ With decades of stroke research focusing on gray matter injury after ischemic stroke, emerging translational studies are highlighting the importance of white matter injury after stroke. White matter is composed mainly of axonal fibers, oligodendrocytes, and other glial cells and is vulnerable to ischemic injury.^2^ Stroke patients enrolled into clinical trials have significant white matter volume changes that occurred during the ischemic process, consisting of approximately 40%‐50% of the stroke volume.^3^ Notably, white matter plays an important role in facilitating neuronal information transmission, thus injury of white matter has been directly associated with development of sensory‐motor dysfunction, paralysis, and cognitive impairments.^4^ Therefore, protecting white matter integrity may represent a potential therapeutic strategy to improve neurological recovery after stroke.

Cerebral ischemic stroke elicits profound activation of the peripheral immune system, leading to influx of immune cells into the ischemic infarct area.^5^, ^6^ Although the pivotal roles of infiltrated immune cells on ischemic brain injury have been well‐documented,^8^, ^9^ their impact on poststroke white matter injury has not been investigated, particularly in scenarios of permanent occlusion model. CD8^+^ T cells are cytotoxic T‐cells activated by foreign antigens presented by professional antigen‐presenting cells and have been suggested to play important roles in demyelinating diseases.^9^, ^13^, ^14^ Cerebral ischemic stroke causes disruption of blood‐brain barrier and leads to leakage of brain‐derived antigen into the periphery,^15^ which may potentially result in profound activation of the antigen‐specific T‐cells, including CD8^+^ T cells.^16^, ^17^ Considering the detrimental role of the cytotoxic CD8^+^ T cells in demyelinating EAE model, the activation and infiltration of CD8^+^ T cells in the ischemic brain may potentially worsen white matter injury after stroke.

CD8^+^ T cells undergo clonal expansion with the help of the cytokine interleukin‐2 (IL‐2),^18^ which is a growth and differentiation factor for T‐cells.^19^ It has recently been shown that IL‐2 exerts dual effects on T‐cell immune responses,^20^ both promoting the generation of effector memory T‐cells and meanwhile maintaining the function of regulatory T‐cells.^21^ Modulation of IL‐2 homeostasis is an important mechanism through which Tregs control CD8^+^ effector differentiation under immunogenic conditions.^22^ Long‐term low dose of IL‐2 improves amyloid pathology in Alzheimer's disease by inducing expansion and activation of regulatory T‐cells in the periphery and brain.^23^ Thus, IL‐2 modulation sheds new light on the treatment of demyelinating injury after stroke.

In this study, we show for the first time that post‐ischemic treatment of IL‐2 monoclonal antibody (IL‐2mAb) JES6‐1 reduces brain infarct volume and preserves white matter integrity in a permanent distal middle cerebral artery occlusion (dMCAO) mouse model. We further demonstrate that posttreatment of IL‐2mAb (JES6‐1) inhibits CD8^+^ T cell activation both centrally and peripherally after ischemic stroke. CD8^+^ T cell depletion abolishes the protection of IL‐2mAb treatment against demyelination, suggesting a causal role of CD8^+^ T cell in IL‐2mAb afforded protection of demyelination after stroke.

## 2 MATERIALS AND METHODS

### 2.1 Animals

Male C57/BL6 mice were purchased from Shanghai SLAC Laboratory Animals and were housed under standard laboratory conditions (22°C and a 12‐hour light‐dark cycle with free access to food and water). All experiments were approved by the Renji Hospital Institutional Animal Care and Use Committee and performed in accordance with the Institutional Guide for the Care and Use of Laboratory Animals.

### 2.2 Experimental design

Male mice were randomly assigned to Sham, MCAO+IgG, and MCAO+IL‐2mAb groups. IL‐2mAb and IgG (eBioscience) were dissolved in PBS before use (10 μg/mL). Animals were administered 0.25 mg/kg IL‐2mAb intraperitoneally 2 and 48 hours after stroke. The dose of IL‐2mAb was determined in the preliminary dose‐response experiment.

### 2.3 Murine focal ischemia model

Male 8‐ to 10‐week‐old C57/BL6 mice were subjected to distal MCAO (dMCAO) or transient MCAO (tMCAO) as previously described.^24^ Briefly, mice were anesthetized with 2% isoflurane in a 30% O_2_/70%N_2_ mixture, and a skin incision was made at the neck and the left CCA was exposed and ligated. After the neck incision was sutured, another skin incision was made between the left eye and ear. The temporal muscle was dissected and a burr hole was opened to expose the distal part of MCA. The dura mater was then cut and the distal MCA was coagulated with low‐intensity bipolar electrocautery (Shanghai Hutong Electronics Co. LTD.) at the immediate lateral part of the rhinal fissure. We induced tMCAO by intraluminal occlusion for the left MCA for 1 hour. Sham‐operated animals underwent anesthesia and surgical exposure of arteries but without artery occlusion. Body temperature was maintained at 37 ± 0.5°C with a heating pad during surgical procedures.

### 2.4 TTC staining and MAP2 staining

We used 2,3,5‐triphenyte tetrazolium chloride staining (TTC staining) and MAP2 to determine the infarct volume. For TTC staining, dMCAO brains were rapidly removed and coronal slices were made at 1 mm from the frontal tips, and sections were immersed in 1.5% TTC (Sigma‐Aldrich) at 37°C for 20 minutes. The presence of infarction was determined by areas on the sides that were not stained with TTC. For MAP2 staining, 25‐μm‐thick coronal sections of MCAO brains were cut every 800 μm and stained with MAP2 antibody (Santa Cruz). We scanned the stained sections and measured the infarct areas using ImageJ. The total infarct volume was obtained by integrating measured infarct areas and distance between sections. Considering the effects of brain edema, subtraction of the ipsilateral minus contralateral hemisphere volume was used for correction.^25^

### 2.5 Neurological function evaluation

The modified Garcia Score,^26^, ^27^ grid walk,^28^ and adhesive test^29^ were performed as previously described to assess sensorimotor functions before and after surgery by investigators who were blinded to experimental group assignments. The modified Garcia Score is a well‐established sensorimotor assessment system consisting of seven individual tests, of which one measures sensor function while four measure motor function. We scored each test from 0 to 3 (maximal score = 15): (a) body proprioception, (b) forelimb walking, (c) limb symmetry, (d) lateral turning, (e) climbing as described. The grid walk test was performed according to Rogers et al with slight modifications. In the grid walk test, mice were placed on an elevated steel grid. Then the foot fault errors (when the animal's forelimb was misplaced and then fell through the grid) were recorded during the moving process. Data are presented as percentage of foot fault errors for the right impaired forelimb referring to the total amount of right forelimb steps. In the adhesive removal test, two adhesive tapes (0.3*0.4 cm^2^) were applied with equal pressure on each animal paw. The order of placement of the adhesive (right or left) was alternated between each animal and each session. Then, the mouse was gently placed in a Perspex box, and the seconds to remove each adhesive tape were recorded. Mice were trained daily before surgery and regularly tested after stroke. Performances of the three final days of each week were averaged as a block.

### 2.6 Immunofluorescence staining and quantification, western blot

Immunostaining was performed on free‐floating 25‐μm sections. Sections were incubated in the following primary antibodies overnight at 4°C: rabbit anti‐Iba1 (Wako), rabbit anti‐CD3 (Abcam), rabbit anti‐myelin basic protein (MBP; Abcam), mouse anti‐nonphosphorylated neurofilament H (SMI32; Calbiochem). Images were captured using a fluorescence microscope (Leica). Immuno‐positive cell counts were presented as the mean number of cells per square millimeter. Three randomly selected microscopic fields within the cortex, striatum, lateral corpus callosum or external capsule on each of three consecutive sections were analyzed for each brain by a blinded investigator. White matter injury was expressed as the mean ratio of SMI32 to MBP immunostaining. The lesion zones of cortex in dMCAO models were studied in more details using Western blot. Myelin basic protein (MBP), oligodendrocyte transcription factor (Olig‐2), CNPase, and oligodendrocyte marker O4 (O4) markers were studied at 7 and 14 days after stroke.

### 2.7 Quantitative real‐time polymerase chain reaction

Quantitative real‐time polymerase chain reaction was performed as previously described. In brief, total RNA was extracted using TRIzol. The first stand of cDNA was synthesized with 1 μg RNA using the PrimeScript^TM^ RT Reagent Kit (Takara). Quantitative real‐time polymerase chain reaction was performed on the LighterCycle 480Ⅱ(Roche) using SYBR green PCR Mix (Yeasen). Primers used are as follows: TNF‐α forward primer: 5‐ ACGGCATGGATCTCAAAGAC‐3, reverse primer: 5‐CGGACTCCGCAAAGTCTAAG‐3, IFN‐γ forward primer: 5‐TGCCAAGTTTGAGGTCAACAACCCA‐3, reverse primer: 5‐ACAGCTGGTGGACCACTCGGA‐3, IL‐6 forward primer: 5‐ACTTCACAAGTCGGAGGCTT‐3, reverse primer 5‐TGCCATTGCACAACTCTTTTCTC‐3, TGF‐β forward primer: 5‐TCGAGGGCGAGAAGTTTA‐3, reverse primer: 5‐AAAAGAATGTCCCGGCTCTC‐3. Expression of GAPDH mRNA served as an internal control.

### 2.8 Flow cytometry

We homogenized the hemisphere ipsilateral to the infarct using a Neural Tissue Dissociation Kit (MACS) by the gentle MACS Dissociator following the manufacturer's instructions (Miltenyi Biotec). The monocyte‐enriched population of cells was collected using a Percoll gradient.^24^ Cells were stained with anti‐mouse CD3, CD4, CD8, CD45, CD25, CD44, CD62L, foxp3, NK1.1, Gr1, and the appropriate isotype controls following manufacturer's instructions (eBioscience), counted on the FACS Verse cell sorter (BD Biosciences) and analyzed using FlowJo software (TreeStar).

### 2.9 In vivo depletion of CD8^+^ T cell

We injected 300 μg of CD8‐specific (clone 53‐6.72; BioXCell) monoclonal antibody or isotype antibody (IgG, clone 2A3; BioXCell) diluted in sterile PBS intraperitoneally 24 hours before ischemia induction. We then confirmed the depletion by flow cytometric analysis of leukocyte subpopulations isolated from blood and spleen.

### 2.10 Statistical analyses

All results are presented as mean±SEM. Student's *t* test was used for two‐group comparisons. For multiple groups, one‐way ANOVA was used followed by the Bonferroni *post hoc* test. Comparisons of means from two groups at multiple time points were performed using two‐way ANOVA when mouse cohorts were different at each time point, and repeated measures ANOVA when the same mice were used throughout the course of the experiment. Results were deemed statistically significant at* P* ≤ 0.05.

## 3 RESULTS

### 3.1 Posttreatment of IL‐2mAb reduces infarct volume and neuroinflammation after stroke

**Figure 1:**
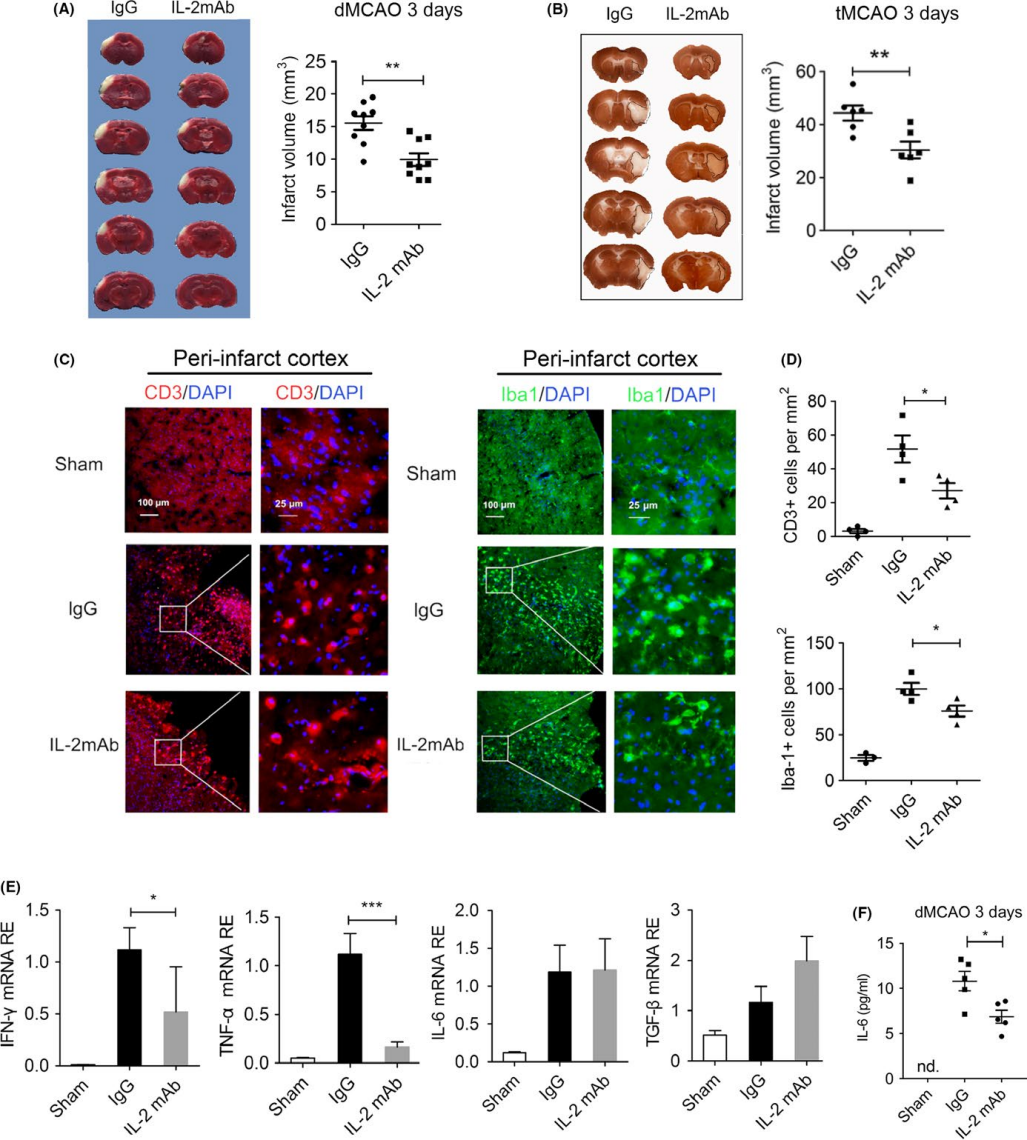
Posttreatment of IL‐2mAb reduces infarct volume and neuroinflammation after stroke. A, TTC staining showed that IL‐2mAb significantly reduced the infarct volume on 3 days after permanent distal middle cerebral artery occlusion (dMCAO). B, MAP2 staining showed similar results in the transient middle cerebral artery occlusion (60‐min tMCAO). C‐D, Representative images and quantitative analysis of infiltrated CD3+ (red) T cells and Iba‐1+ (green) microglia of the peri‐infarct cortex in IgG and IL‐2mAb group. IL‐2mAb significantly decreased the infiltrated numbers 3 days after dMCAO. E, RT‐RCR showed that the relative expression of pro‐inflammatory cytokines including IFN‐γ and TNF‐α were decreased in IL‐2mAb group 3 days after dMCAO. F, ELISA showed decreased expression level of IL‐6 of the blood in IL‐2mAb group. Data are expressed as mean±SEM. *P ≤ 0.05, **P ≤ 0.01, ***P ≤ 0.001 vs IgG. Student's *t* test, one‐way ANOVA, Bonferroni *post hoc* test. n = 5‐8 per group

Previous studies indicated that ischemic stroke increases IL‐2 levels in the periphery.^30^ Because IL‐2 plays a critical role in regulating CD8^+^ T cell activation, and CD8^+^ T cell activation is detrimental for myelinated neural fibers, we hypothesized that neutralization of IL‐2 with IL‐2mAb may be protective against demyelination in the late phase of cerebral ischemic injury. First, we tested the effect of IL‐2mAb on the brain infarct after ischemic stroke. We injected IL‐2mAb at 2 and 48 hours after dMCAO. We found that IL‐2mAb significantly reduced the infarct volume on 3 days after permanent distal middle cerebral artery occlusion compared with the IgG‐treated group (15.54 ± 3.22 and 8.2 ± 2.39, *P* < 0.01, Figure 1A). We found similar results in the 60‐minutes tMCAO model (44.34 ± 6.94 and 30.30 ± 7.94, *P* < 0.01, Figure 1B) 3 days after surgery. We next investigated the role of IL‐2mAb treatment on poststroke neuroinflammation by measuring the infiltration of peripheral pro‐inflammatory cells and the activation of resident microglia. Consistent with the lesion size, IL‐2mAb decreased CD3^+^ T cells and Iba1^+^ microglia in the ischemic penumbra (*P* < 0.05, Figure 1C,D). In addition, we found IL‐2mAb treatment significantly reduced the mRNA expression of pro‐inflammatory cytokines in the ischemic brain, such as IFN‐γ and TNF‐α (*P* < 0.05, *P* < 0.001, respectively, Figure 1E), but slightly increased the mRNA expression of TGF‐β, and left the IL‐6 level unchanged (Figure 1E). The pro‐inflammatory cytokine IL‐6 in the peripheral blood measured by ELISA was decreased in the IL‐2mAb group (Figure 1F). The above results suggest that IL‐2mAb treatment reduces infarct volume and inhibits neuroinflammatory response in the ischemic brain after stroke.

### 3.2 IL‐2mAb improves long‐term functional recovery of permanent distal MCAO mice

**Figure 2:**
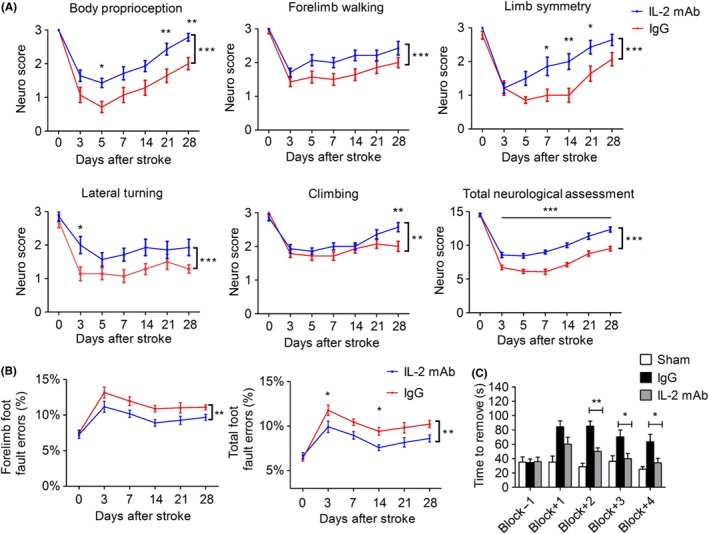
IL‐2mAb improves long‐term functional recovery of permanent distal MCAO mice. A‐C, Sensorimotor dysfunction was significantly attenuated in IL‐2mAb‐treated mice ≤28 days after ischemia, as assessed by the Garcia Score (A), grid walk (B) and adhesive remove (C) test. A, IL‐2mAb‐treated mice demonstrated improved scores in body proprioception, limb symmetry, lateral turning, climbing, and total assessment. B‐C, Less sensor‐motor deficits were observed in the IL‐2mAb group as reflected by less total foot fault and shorter removal time of the sticker. Data are expressed as mean±SEM. *P ≤ 0.05,**P ≤ 0.01, ***P ≤ 0.001 vs IgG. Two‐way repeated measures ANOVA for differences between groups. n = 10‐12 per group

We next examined the effect of IL‐2mAb on the long‐term functional recovery after permanent dMCAO using three different behavioral tests, including modified Garcia score, grid‐walking test, and adhesive removal test. IL‐2mAb‐treated mice exhibited significantly higher scores in body proprioception, climbing, and total score than the IgG‐treated control mice up to 28 days after stroke (*P* < 0.01, *P* < 0.01, *P* < 0.001, respectively, Figure 2A). In the grid‐walking test, IL‐2mAb‐treated mice also made fewer total foot faults at 3 and 14 days after stroke (*P* < 0.05, Figure 2B). In the adhesive removal test, IL‐2mAb‐treated mice required shorter time to remove the stickers placed on their paws than IgG‐treated control mice at 14, 21, and 28 days after stroke (*P* < 0.01, *P* < 0.05, *P* < 0.05, respectively, Figure 2C). Collectively, these results support the concept that posttreatment of IL‐2mAb improves long‐term functional recovery after permanent MCAO.

### 3.3 IL‐2mAb reduces demyelination in the late phase of permanent distal ischemic stroke

**Figure 3:**
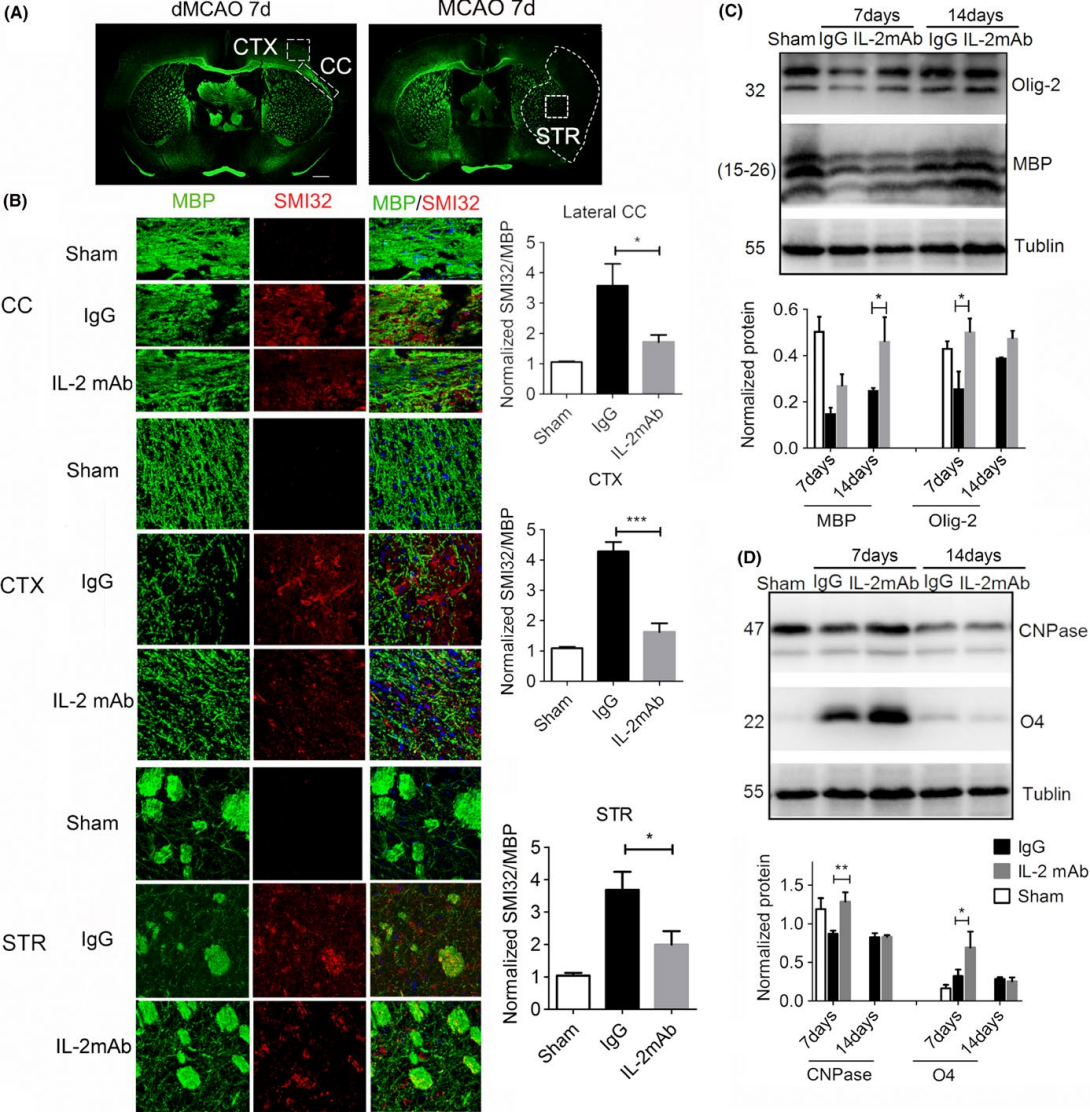
IL‐2mAb reduces the demyelination in the late phase of permanent distal ischemic stroke. A, Representative imaging area from dMCAO (left) and tMCAO (right) mice brain. B, Representative images of increased the expression of MBP (green) and decreased the expression of SMI32 (red) 7 days after dMCAO and tMCAO in the IL‐2mAb treated group. Upper panel is lateral corpus callosum, middle panel is peri‐infarct cortex in dMCAO model, and bottom panel is striatum in tMCAO model. C, Western blot showed that IL‐2mAb significantly increased the expression of myelin differentiation‐associated markers (MBP) in the lesion area 14 days after dMCAO. D, Western blot showed that IL‐2mAb significantly increased the expression of OPC markers (CNPase, O4) 7 days after dMCAO. Data are expressed as mean±SEM. *P ≤ 0.05 vs IgG. One‐way ANOVA, Bonferroni *post hoc* test. n = 5 per group

Because white matter integrity is closely associated with long‐term sensory motor function and cognitive function in cerebral ischemic stroke,^4^ and demyelination is an important component of white matter injury, we next examined myelination of axonal fibers in the ischemic brain using immunofluorescent double labeling for myelin basic protein (MBP), a marker of myelin integrity, and nonphosphorylated neurofilament H (SMI32), a marker for axonal damage.^31^ The left panel of Figure 3A illustrated the peri‐infarct cortex, lateral corpus callosum area where the images were taken in the brain sections of dMCAO mice, while the right one is the striatum in the tMCAO model. Loss of MBP immunoreactivity was detected accompanied by increase in SMI32 expression in the above‐mentioned areas 7 days after stroke compared with the sham group (Figure 3B). IL‐2mAb treatment attenuated both MBP loss and SMI‐32 increase in the ischemic cortex and protected the integrity of the lateral corpus callosum at 7 days after stroke in dMCAO model (Figure 3B). IL‐2mAb therapy also attenuated the white matter injury in the striatum of tMCAO model 7 days after stroke (Figure 3B). Western blot confirmed the expression changes revealed by immunofluorescent staining. At 7 and 14 days after stroke, we analyzed the expression level of white matter differentiation related markers (MBP, Olig2) and oligodendrocyte progenitor cell markers (CNPase, O4) in the lesion cortex of dMCAO mice. MBP was significantly increased in IL‐2mAb‐treated mice compared to control mice at 14 days after ischemia (*P* < 0.05, Figure 3C). Likewise, Olig‐2, CNPase, and O4 were increased at 7 days after stroke in IL‐2mAb‐treated mice (*P* < 0.05, *P* < 0.01, *P* < 0.05, respectively, Figure 3C, D). Compared to 7 days after stroke, the levels of OPC markers at 14 days were not significantly changed between IgG and IL‐2 treated groups (Figure 3C,D). To exclude the direct effect of IL‐2mAb on myelination, we also tested the expression of MBP, Olig‐2, CNPase with intraperitoneal injection of IL‐2mAb in normal mice and found that IL‐2mAb did not significantly change the expression of the above proteins at 7 and 14 days after injection, but only increased O4 expression at 7 days after injection, which returned to normal at 14 days (Figure S1).

These data suggest that IL‐2mAb treatment attenuates the demyelination process after stroke.

### 3.4 Brain infiltration of T‐cells, in particular CD8^+^ T cells, were suppressed in IL‐2mAb‐treated mice

**Figure 4:**
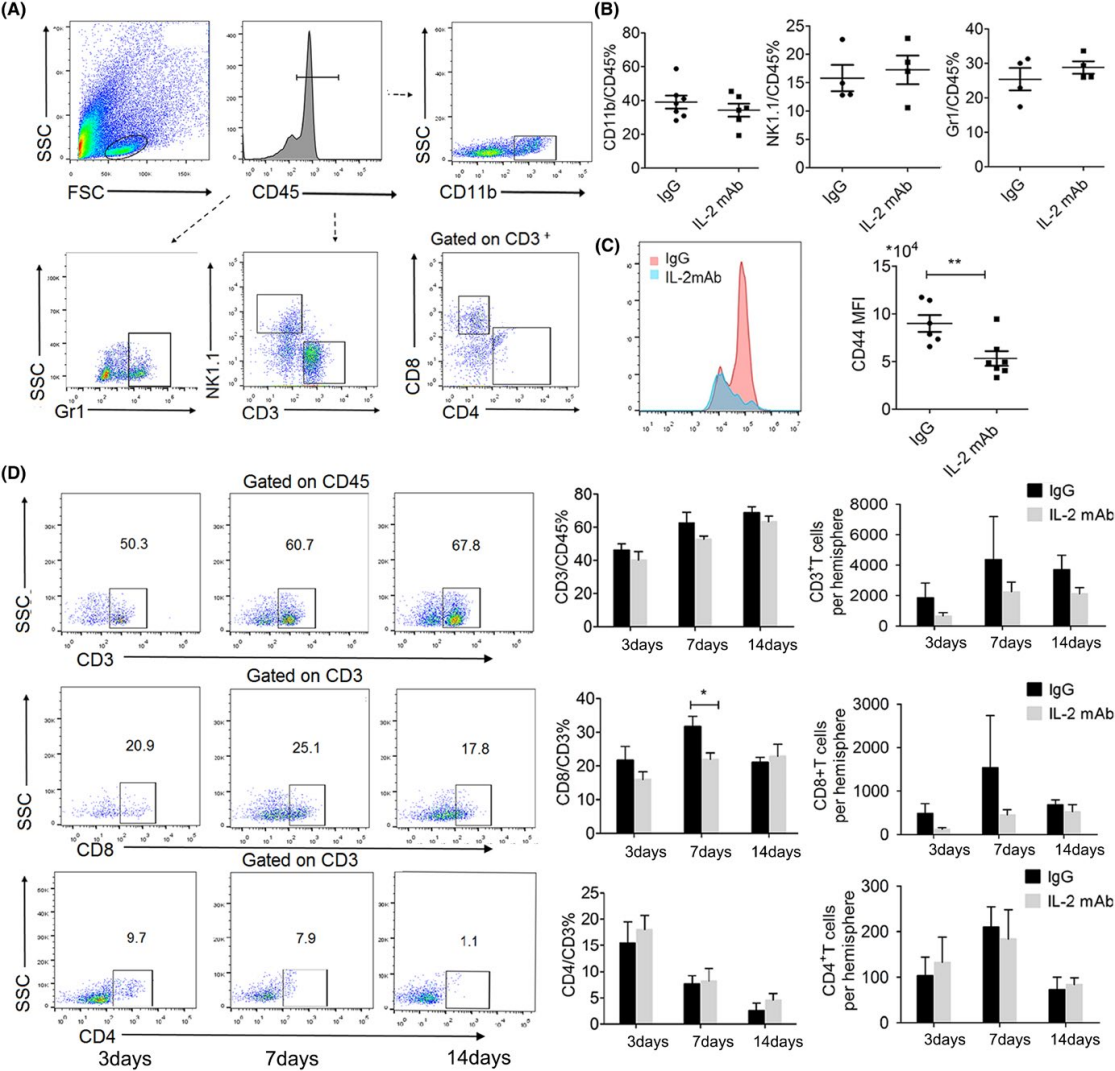
Brain infiltration of T cells, in particular CD8+ T cells, was suppressed in IL‐2mAb‐treated mice. A. Gating strategy for flow cytometric analysis of brain‐invading leukocyte subsets. Viable leukocytes were gated on scatter gram followed by gating for CD45+ leukocytes and additional analysis of subpopulations as indicated: CD3+, CD4+, CD8+, NK1.1+, Gr1+, and CD11b+ cells. B, IgG and IL‐2mAb group showed similar amount of CD11b+, Cr1+neutrophils NK1.1 + natural killer cells. C, CD44 MFI of CD8+ T cell significantly reduced 7 days after dMCAO. D, Representative flow cytometric plots and quantitative analysis of the brain infiltration pattern of CD3+T cell, CD8+ T cell, and CD4+ T cell after dMCAO up to 14 days in IgG and IL‐2mAb‐treated mice. IL‐2mAb reduced the CD8+/CD3+T cells 7 days after dMCAO. Data are expressed as mean±SEM. *P ≤ 0.05 and **P ≤ 0.01 vs IgG. One‐way ANOVA, Bonferroni post hoc test. n = 5‐6 per group

Stroke elicits profound immune responses in both periphery and brain, which have been shown to play critical roles in aggravating ischemic brain injury and lymphocytes are one of the major cell types that affects ischemic brain injury..^32^ IL‐2 is a critical regulator of lymphocyte homeostasis and function, as well as a powerful stimulant for T lymphocytes and NK cells.^19^ Accordingly, we isolated brain‐invading leukocytes from the hemisphere of IgG and IL‐2mAb‐treated mice 7 days after dMCAO and analyzed leukocyte subsets by flow cytometry (Figure 4A). We found that the percentage of CD11b^+^/CD45^+^ cells, NK1.1^+^/CD45^+^ cells, and Gr1^+^/CD45^+^ cells were not significantly changed in IgG or IL‐2mAb treated groups (Figure 4B). However, CD8^+^/CD45^+^ cells in the ischemic brain were decreased in the IL‐2mAb treated stroke brain, while the MFI of CD44 on CD8^+^ T cells was significantly reduced (*P < *0.01, Figure 4C). We further compared the effect of IL‐2mAb on different T lymphocyte subpopulations (CD3^+^ T cells, CD4^+^ T cells, CD8^+^ T cells) at different time points in the ischemic brain after stroke. We observed a trend toward increased infiltration of CD3^+^ T cell into the ischemic brain 3 days after stroke, and it was further increased 14 days after stroke (Figure 4D). A large amount of CD8^+^/CD3^+^ T cells (18.26% ± 3.43%) were still detectable 14 days after stroke (Figure 4D), showing consistent time frame with the temporal pattern of delayed white matter demyelination. Unlike CD8^+^ T cells, however, CD4^+^ T cells showed different pattern of infiltration, rising prominently 3 days after stroke, and then decreasing 7 days after stroke, to the point that only a small proportion of the gated cells were detectable as CD4^+^ T cells 14 days after stroke (Figure 4D). IL‐2mAb treatment slightly reduced the infiltration of CD3^+^ T cells, but significantly reduced the proportion of CD8^+^ T cells among CD3^+^ T cells in the ischemic brain 7 days after stroke (*P* < 0.05, Figure 4D). However, IL‐2mAb did not change CD8^+^ T cells infiltration significantly 14 days after stroke. For CD4^+^ T cells' infiltration, IL‐2mAb treatment did not make significant changes at any of the three time points (Figure 4D). Due to the rare infiltration of lymphocytes in the sham hemisphere (Figure S2), we did not show quantitative data from the sham mice. These results suggest that IL‐2mAb treatment suppresses the infiltration of CD8^+^ T cells into the ischemic brain, in a time frame similar to IL‐2mAb‐afforded protection of white matter integrity in the ischemic brain.

### 3.5 IL‐2mAb treatment inhibits peripheral CD8^+^ T cell activation during the acute phase of stroke

**Figure 5:**
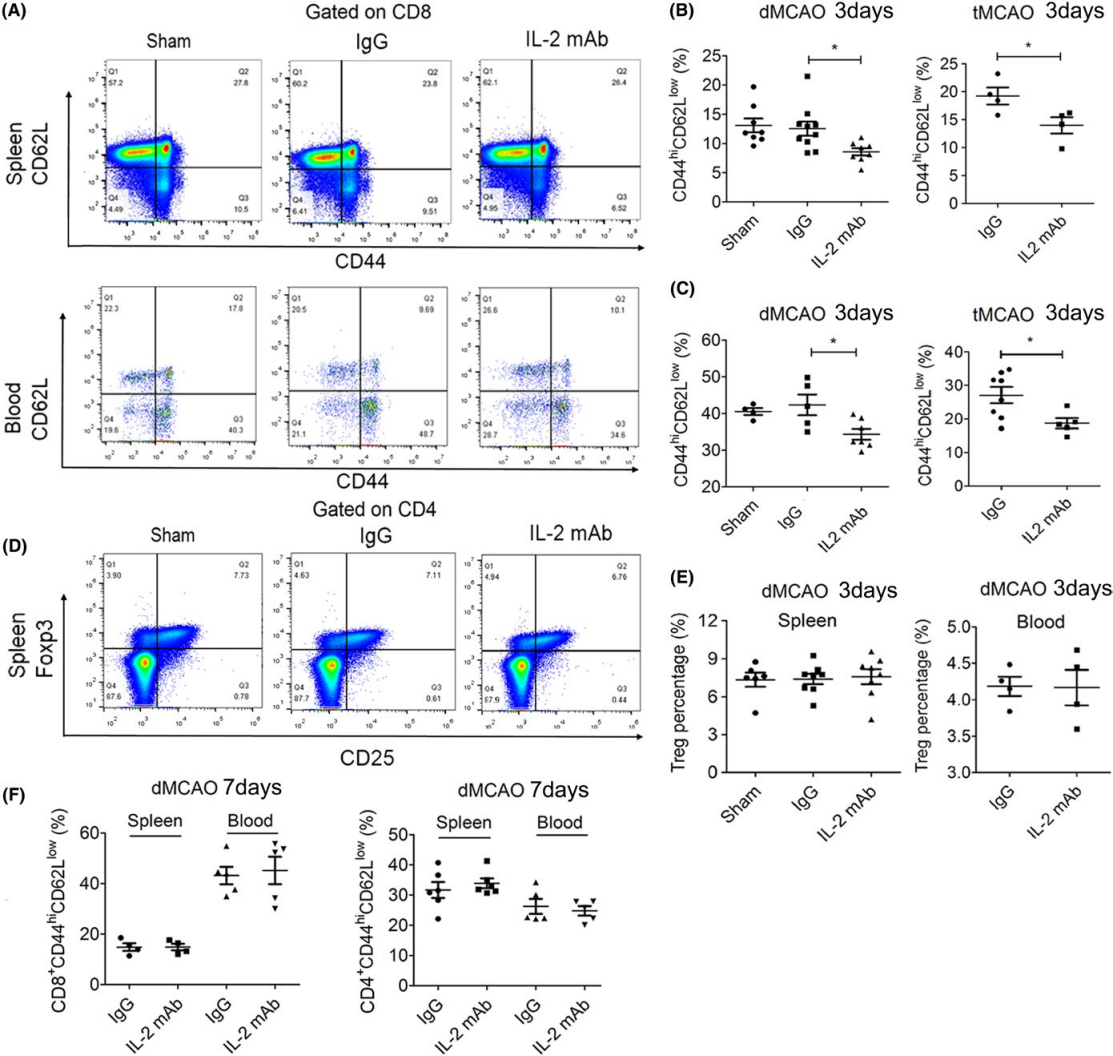
IL‐2mAb treatment inhibits peripheral CD8+ T cell activation during the acute phase of stroke. A‐B, The percentage of CD8+CD44hiCD62Llo in the spleen and peripheral blood was significantly reduced by the IL‐2mAb treatment 3 days after in both dMCAO and tMCAO (n = 6‐9 per group). C‐D, The percentage of Tregs in the spleen and blood was unchanged by IL‐2mAb treatment 3 days after dMCAO. E. The percentage of CD8+CD44hiCD62Llo and CD4+CD44hiCD62Llo in the spleen and blood was not changed by IL‐2mAb treatment 7 days after dMCAO (n = 4‐6 per group). Data are expressed as mean±SEM. *P ≤ 0.05 vs IgG. One‐way ANOVA, Bonferroni *post hoc* test

In order to investigate the peripheral immune changes after IL‐2mAb treatment, we found that effector memory T‐cells (CD8^+^CD44^hi^CD62L^lo^T cells), which reflects the activation state of CD8^+^ T cells, were significantly decreased in the spleen and peripheral blood of IL‐2mAb‐treated mice compared to the IgG control mice 3 days both after dMCAO and tMCAO (12.56 ± 3.80 vs 8.57 ± 1.74, 42.34 ± 6.34 vs 34.33 ± 3.91, *P* < 0.05, respectively, Figure 5A‐C). However, IL‐2mAb treatment did not lead to significant changes in the percentage of CD4^+^CD44^hi^CD62L^lo^ T cells or regulatory T‐cells in the periphery at 7 days after stroke (Figure 5D‐F). The activation state of both CD8^+^ T cell and CD4^+^ T cell showed no difference between these two groups 7 days after dMCAO (Figure 5F). The results suggest that IL‐2mAb posttreatment may modulate the activation of CD8^+^ T cells in the peripheral during the acute phase of stroke.

### 3.6 CD8^+^ T cells are critical for IL‐2mAb afforded protection of white matter injury after stroke

**Figure 6:**
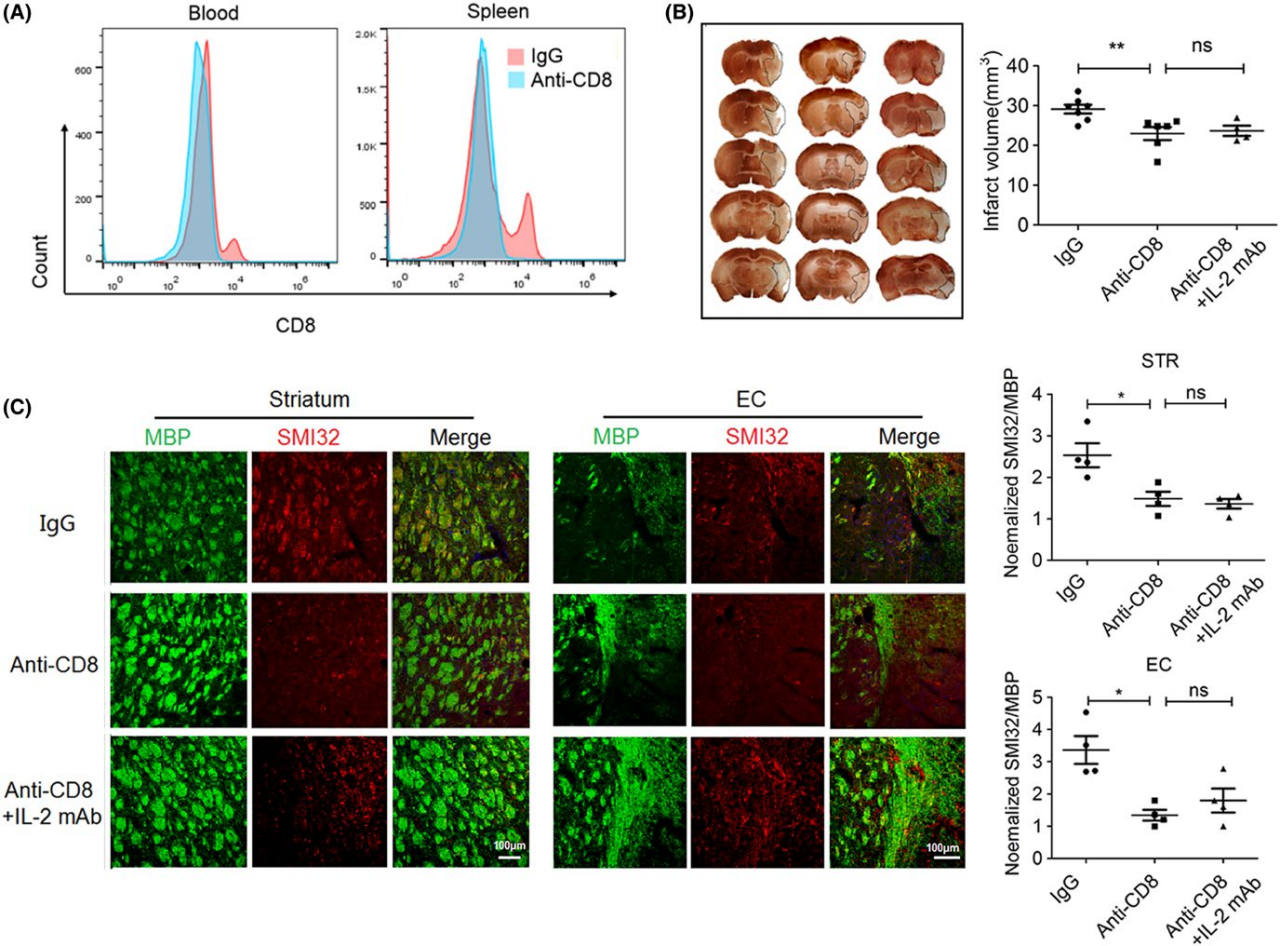
CD8+ T cells are critical for IL‐2mAb afforded protection of white matter injury after stroke. A. Representative flow cytometric plots validated the effectiveness of the ablation of CD8+ T cells. B. MAP2 staining showed that ablation of CD8+ T cells significantly reduced the infarct volume 7 days after tMCAO, while ablation of CD8+ T cells combined with IL‐2mAb treatment did not further reduce the infarct volume, similar to CD8+ T cell ablation group (n = 5‐7 per group). C. Representative images showed that ablation of CD8+ T cells alleviated the demyelination injury in the striatum and lateral corpus callosum region in tMCAO, including the increased immunofluorescence intensity of MBP, and decreased intensity of SMI32. CD8+ T cells ablation combined with IL‐2mAb treatment did not further reduce the extent of demyelination injury, showing similar immunofluorescence of MBP and SMI32 compared with CD8+ T cell ablation group. Data are expressed as mean±SEM. *P ≤ 0.05 and **P ≤ 0.01 vs IgG. ns, not significant. One‐way ANOVA, Bonferroni *post hoc* test

In order to investigate whether the suppression of CD8^+^ T cells is causally related to the preservation of white matter integrity after IL‐2mAb treatment, we depleted CD8^+^ T cells by injecting the anti‐CD8 antibody 24 hours before MCAO surgery. Using flow cytometry, we confirmed that a single intraperitoneal injection of CD8 neutralization antibody completely eliminated the CD8^+^ T cell population (Figure 6A). CD8^+^ T cell‐depleted mice exhibited reduced infarct volume compared to non‐depleted (IgG‐only injected) ischemic mice (22.98 ± 4.00 vs 29.11 ± 2.92,* P* < 0.01) 7 days after stroke (Figure 6B) and protected against the damage to white fiber bundles in the striatum and external capsule (Figure 6B, C). These results indicate that CD8^+^ T cells have a detrimental role in the progression of white matter injury. We then injected IL‐2mAb to the CD8^+^ T cell‐depleted mice and found that IL‐2mAb treatment did not provide further reduction of infarct volume (22.98 ± 4.00 and 23.67 ± 2.56, *P* > 0.05, Figure 6B) or protection of white matter integrity in the CD8^+^ T cell‐depleted mice (Figure 6C). Collectively, these data suggest that IL‐2mAb (JES6‐1) reduces cerebral infarct volume and alleviates demyelinating injury through attenuating CD8^+^ T cell activation.

## 4 DISCUSSION

Recent studies highlight the importance of white matter injury in the long‐term functional outcome of ischemic stroke.^33^, ^34^ Severe white matter lesions have been suggested as a prognostic factor for poor activities of daily living at discharge in elderly stroke patients.^35^ To the best of our knowledge, the present study provides the first evidence that posttreatment of IL‐2mAb (JES6‐1) preserves poststroke white matter integrity and improves functional recovery up to 28 days after stroke. Another major finding of this study is that brain infiltrating CD8^+^ T cells possess detrimental actions in poststroke white matter injury. We have shown that blocking the activation and brain infiltration of CD8^+^ T cells using IL‐2mAb (JES6‐1) preserve white matter integrity in both dMCAO and 60‐minutes tMCAO. Thus, we propose that IL‐2mAb (JES6‐1) treatment may serve as a novel therapeutic strategy to improve long‐term neurological functions after stroke.

Oligodendrocytes, which are responsible for myelination of neuronal axons, can also be damaged by ischemic and hypoxic insults along with neurons.^4^ Importantly, white matter injury after stroke tends to persist longer than gray matter injury.^36^, ^37^ Consistent with previous studies,^36^ we also found prominent white matter injury at the later phase of stroke recovery (Figure 3). Therefore, preserving the integrity of white matter may represent a promising strategy to improve the long‐term neurological outcomes of ischemic stroke. An important finding of the current study is that IL‐2mAb posttreatment reduced the white matter demyelinating injury and improved neurological performances of experimental stroke mice up to 28 days after ischemic stroke.

The above protection of white matter may be closely related to the suppression of the poststroke adaptive immune system. Stroke elicits profound activation of peripheral immune system and infiltration of peripheral immune cells into the brain.^5^ Acute immune interventions that avoid over‐activation of peripheral immune cells reduce immune cell‐mediated neuronal cell injury and attenuate subsequent poststroke immunosuppression.^32^ Adaptive immune CD8^+^ T cells have been suggested to play cardinal roles in both stroke and many autoimmune and inflammatory demyelinating diseases, such as multiple sclerosis (MS) and Rasmussen's encephalitis.^4^, ^13^, ^38^ One of the hallmarks of MS lesions is large infiltrates of immune cells exist in the central nervous system. Brain sections of clinical MS patients revealed more abundant CD8^+^ T cells than CD4^+^ T cells in the demyelinating lesions.^39^ In the white matter regions adjacent to MS lesions, the predominant group of infiltrating cells is CD8^+^ T cells, while the CD4^+^ T cells are more likely inclined to the perivascular location.^39^, ^40^ Similarly, both our study and previous research reported profound activation and infiltration of CD8^+^ T cells in the late phase of ischemic stroke.^24^ Under a neuro‐inflammatory environment, the expression of MHC‐1 in the resident astrocytes, oligodendrocytes, axons, and neurons was upregulated, and thus all these cells become a target of CD8^+^ T cells.^41^ Thus, the infiltrating CD8^+^ T cells after stroke are also likely to mediate poststroke white matter injury and worsen neurological deficits. In addition, decreasing infiltration of CD8^+^ T cells in the central nervous system via blocking α4 integrin with myelin oligodendrocyte glycoprotein (MOG33‐35) protects the white matter damage in the EAE model.^42^ Similarly, blocking the activation and infiltration of CD8^+^ T cells may reverse the demyelinating injuries during the late phase of ischemic stroke recovery.

IL‐2 is a cytokine that supports growth, differentiation, and clonal expansion of T‐cells 19 and plays a major role in CD8^+^ T cell programming. Prolonged IL‐2 signaling promotes terminal‐effector differentiation in vivo.^43^ IL‐2 levels are elevated in serum samples from stroke patients taken 24 hours after stroke onset and are sustained in severe patients.^30^ Daclizumab, a humanized monoclonal antibody directed against high‐affinity IL‐2R and modulates IL‐2 signaling, shows a high efficacy in reducing immune pathogenesis of MS.^44^ Therefore, neutralizing IL‐2 after stroke is likely to block the activation of CD8^+^ T cells and protects against demyelinating injuries after stroke.

Our in vivo experiments demonstrate that the IL‐2mAb treatment poststroke leads to the decrease of peripheral CD8^+^ T activation during the acute phase without any changes to CD4^+^ T cells and Tregs. The infiltrating CD8^+^ T cells into the ischemic brain were also less activated in the IL‐2mAb treated group, whereas the infiltration of neutrophils, B cells, and macrophages was left unchanged (Figures 4, 5). These results suggested that IL‐2mAb treatment reduced the cytotoxic effect of CD8^+^ T cells in the ischemic brain. We further analyzed white matter integrity in both groups and found that IL‐2mAb posttreatment significantly reduced demyelinating injury 14 days after ischemic stroke (Figure 3). In order to test the causal relationship between CD8^+^ T cells in the IL‐2mAb afforded protection of white matter integrity, we depleted CD8^+^ T cells using CD8‐specific monoclonal antibody 24 hours before IL‐2mAb treatment. We made a novel finding that in the absence of CD8^+^ T cells, IL‐2mAb (JES6‐1) could not provide further protection against demyelination after ischemic stroke (Figure 6B,C).

This study has several pitfalls. Although we have shown that IL‐2mAb (JES6‐1) reduces white matter demyelination, it remains unknown whether IL‐2mAb affects poststroke remyelination or simply prevents demyelination directly. It is also likely that, by suppressing CD8^+^ T cells, IL‐2mAb also promotes the myelin repair process and eventually leads to sustained white matter injury after stroke. We proposed that CD8^+^ T cells played a causal role in IL‐2mAb exerted protection of poststroke white matter integrity, but the underlying mechanism by which CD8^+^ T cells affect the white matter has not been investigated. Future studies to address the potential underlying mechanisms would be highly interesting and likely lead to new targets for therapeutic intervention in stroke recovery.

## 5 CONCLUSION

In conclusion, our findings shed new light on the pivotal role of CD8^+^ T cells in the white matter injury after cerebral ischemic stroke and we propose that IL‐2mAb may be an intriguing therapeutic strategy for long‐term neuroprotection against demyelination after stroke.

## CONFLICT OF INTEREST

Dr Stetler receives moderate to significant funding from the National Institutes of Health. However, those projects did not provide the funding for this study. The other authors report no conflicts.
