# Supplementary material for: IL‐2mAb reduces demyelination after focal cerebral ischemia by suppressing CD8+ T cells
Source: CNS Neurosci Ther. 2018 Nov 15;25(4):532–43. doi: 10.1111/cns.13084 (PMC6488908; doi:10.1111/cns.13084)
Supplement: Supplementary file 1 [file CNS-25-532-s001.docx]

Supplement Figures

Supp Fig 1. The effects of IL-2mAb treatment on expression of myelin associated proteins under physical condition.


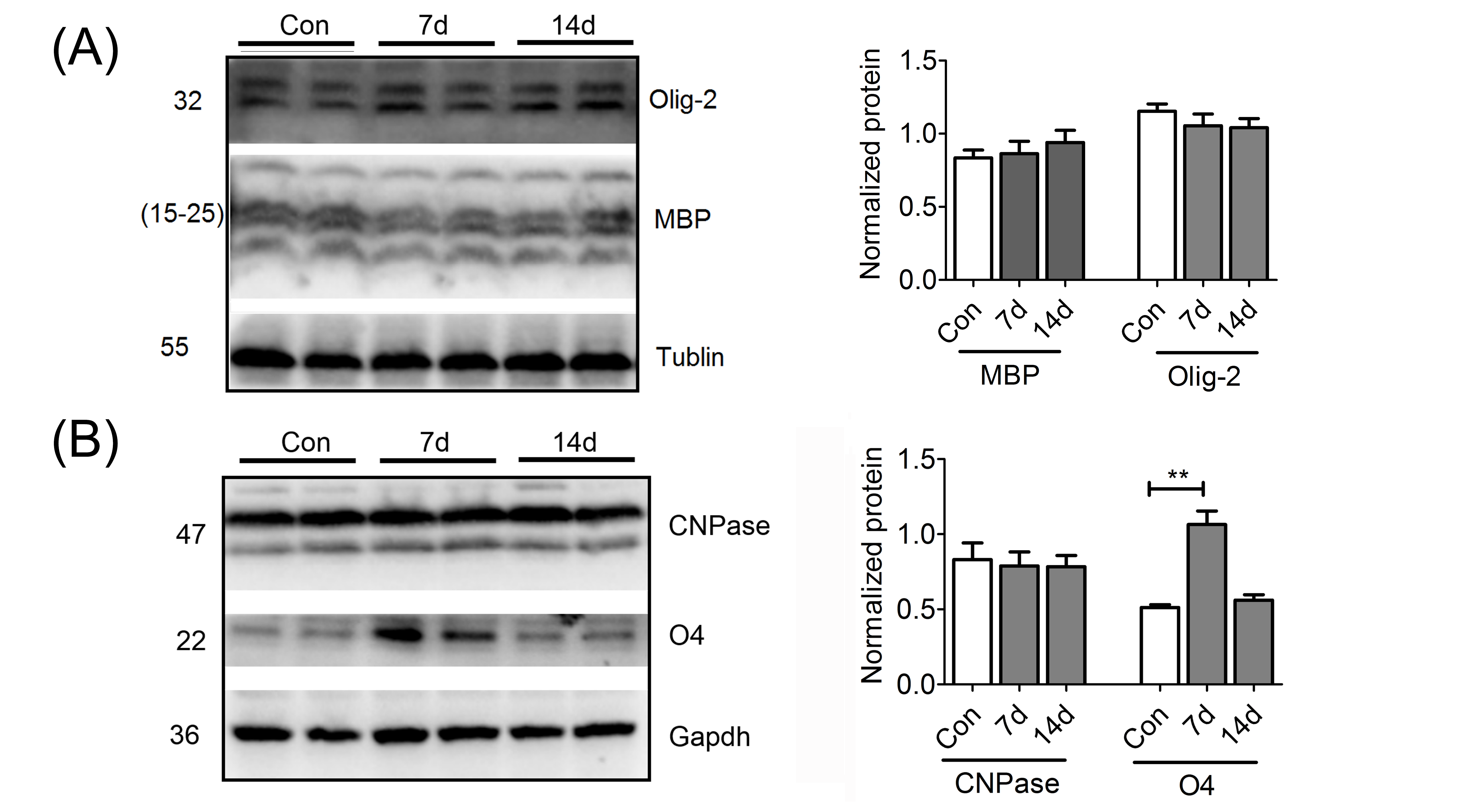


A. Intraperitoneal injection of IL-2 mAb has no effect on the expression of MBP, olig-2 in the cortex of normal mice. B. The expression of CNPase is left unchanged after IL-2mAb injection. IL-2mAb increased the expression of O4 7 days, but not 14 days after injection.

Supp Fig 2. Brain infiltration of immune cells in sham group.


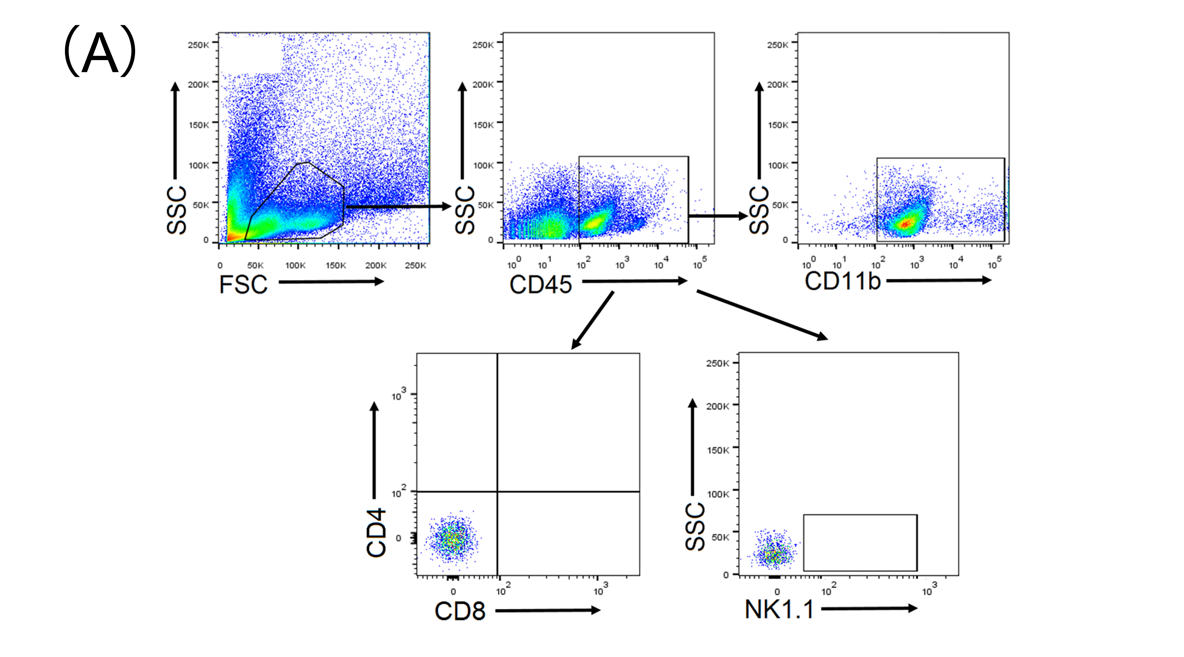


A. Gating strategy for flow cytometric analysis of brain-invading leukocyte subsets in sham hemisphere.
